# Reply: Clinical Context in Artificial Intelligence-Enabled Electrocardiographic Risk Stratification for Atrial Fibrillation

**DOI:** 10.1016/j.jacadv.2026.103313

**Published:** 2026-10-07

**Authors:** Yoshihiro Sobue, Kazuya Takeda, Kouki Matsuo, Eiichi Watanabe, Hideo Izawa

**Affiliations:** a Department of Cardiology, Fujita Health University Bantane Hospital, Nagoya, Japan; b Graduate School of Health Sciences, Fujita Health University Graduate School, Toyoake, Japan; c Department of Cardiology, Fujita Health University, Toyoake, Japan

We thank Drs Sato and Takahashi for their thoughtful comments on our study.^1^ Their observations highlight an important aspect of artificial intelligence-enabled electrocardiography (AI-ECG): AI-ECG likelihood should not be interpreted simply as a surrogate for the presence or absence of atrial fibrillation (AF).

Some patients with documented AF may have lower AI-ECG likelihood because the algorithm evaluates subtle sinus-rhythm ECG features associated with atrial electrical remodeling rather than AF history itself.^1^ Thus, lower AI-ECG likelihood in established AF may reflect less advanced or less ECG-evident electrical remodeling. In our cohort, higher AI-ECG categories were accompanied by progressively greater left atrial dimensions, supporting an association with the remodeling phenotype. The inverse associations of hypertension and left ventricular mass index with AI-ECG likelihood reported by Sato et al^2^ are intriguing. Differences in sample size, patient selection, and clinical phenotype between their 104 patients with documented AF and our broader real-world cohort of 665 patients may partly explain these apparently divergent findings.

We agree that correlated predictors may influence SHapley Additive exPlanations feature attribution. Our original supplementary analyses evaluated SHapley Additive exPlanations both without and with CHADS_2_ and CHA_2_DS_2_-VASc.^1^ To further address predictor redundancy, we performed an additional post hoc sensitivity analysis in the external validation cohort using a reduced predictor set that retained CHA_2_DS_2_-VASc while excluding its overlapping individual components (age, sex, hypertension, diabetes, heart failure, prior stroke/transient ischemic attack, and vascular disease). As shown in Figure 1, CHA_2_DS_2_-VASc remained among the 3 most influential features across classifiers, while feature attribution was distributed across other clinical, laboratory, and structural variables, including hemoglobin and N-terminal pro–B-type natriuretic peptide (NT-proBNP). Importantly, AI-ECG retained a modest contribution across classifiers, supporting its complementary role alongside conventional clinical information.

**Figure 1:**
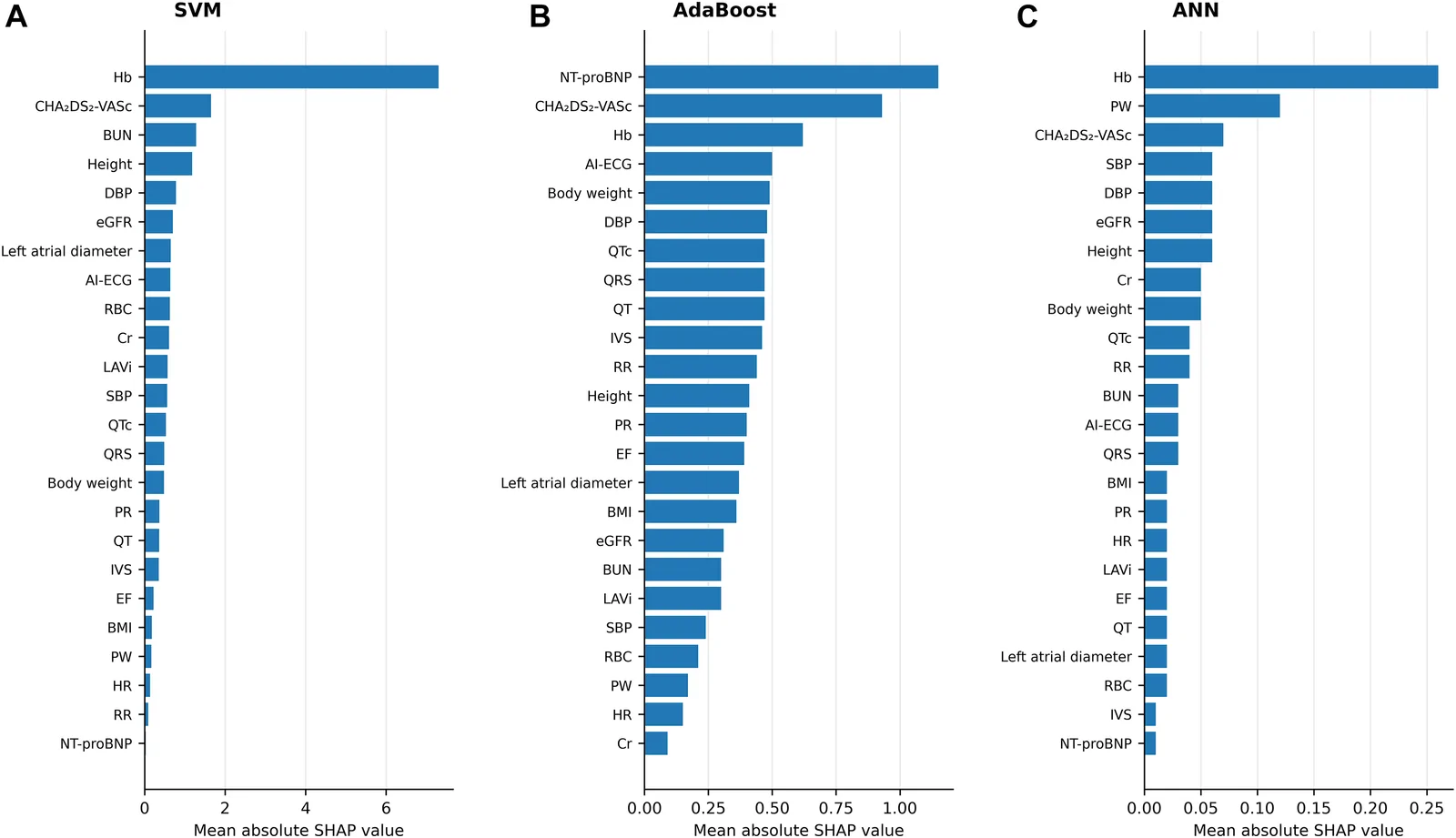
SHapley Additive ExPlanations Feature Attribution Using a Reduced Predictor Set Post hoc sensitivity analysis in the external validation cohort using a reduced predictor set retaining CHA_2_DS_2_-VASc while excluding its overlapping individual components (age, sex, hypertension, diabetes, heart failure, prior stroke/transient ischemic attack, and vascular disease). Mean absolute SHapley Additive exPlanations values are shown across the 3 classifiers: (A) support vector machine (SVM), (B) adaptive boosting (AdaBoost), and (C) artificial neural network (ANN). AI-ECG = artificial intelligence–enabled electrocardiography; ANN = artificial neural network; BMI = body mass index; BUN = blood urea nitrogen; Cr = creatinine; DBP = diastolic blood pressure; EF = ejection fraction; eGFR = estimated glomerular filtration rate; Hb = hemoglobin; HR = heart rate; IVS = interventricular septal thickness; LAVi = left atrial volume index; NT-proBNP = N-terminal pro–B-type natriuretic peptide; PR = PR interval; PW = posterior wall thickness; QT = QT interval; QTc = corrected QT interval; QRS = QRS duration; RBC = red blood cell count; RR = RR interval; SBP = systolic blood pressure; SHAP = SHapley Additive exPlanations; SVM = support vector machine.

Our take-home message therefore remains unchanged: AI-ECG provides complementary information for AF discrimination but should be interpreted alongside established clinical and structural characteristics rather than as a replacement for conventional clinical assessment.
